# Supplementary material for: Autoencoder neural networks enable low dimensional structure analyses of microbial growth dynamics
Source: Nat Commun. 2023 Dec 1;14:7937. doi: 10.1038/s41467-023-43455-0 (PMC10696002; doi:10.1038/s41467-023-43455-0)
Supplement: Supplementary file 1 — Supplementary Information [file 41467_2023_43455_MOESM1_ESM.pdf]

Supplementary Information

Supplementary Figures 1-7

Supplementary Table

Supplementary Methods

Autoencoder neural networks enable low dimensional structure analyses of microbial growth dynamics

Yasa Baig[1,2], Helena R. Ma[3,4], Helen Xu[2], Lingchong You[3,4,5*]

[1]Department of Physics, Duke University, Durham, NC, United States;

[2]Department of Computer Science, Duke University, Durham, NC, United States;

[3]Department of Biomedical Engineering, Duke University, Durham, NC, United States;

[4]Center for Quantitative Biodesign, Duke University, Durham, NC, United States;

[5]Department of Molecular Genetics and Microbiology, Duke University School of Medicine, Durham, NC, United States

*Correspondence and requests for materials should be addressed to Lingchong You. E-mail: you@duke.edu; Tel: 919-660-8408; Fax: 919-668-0795.

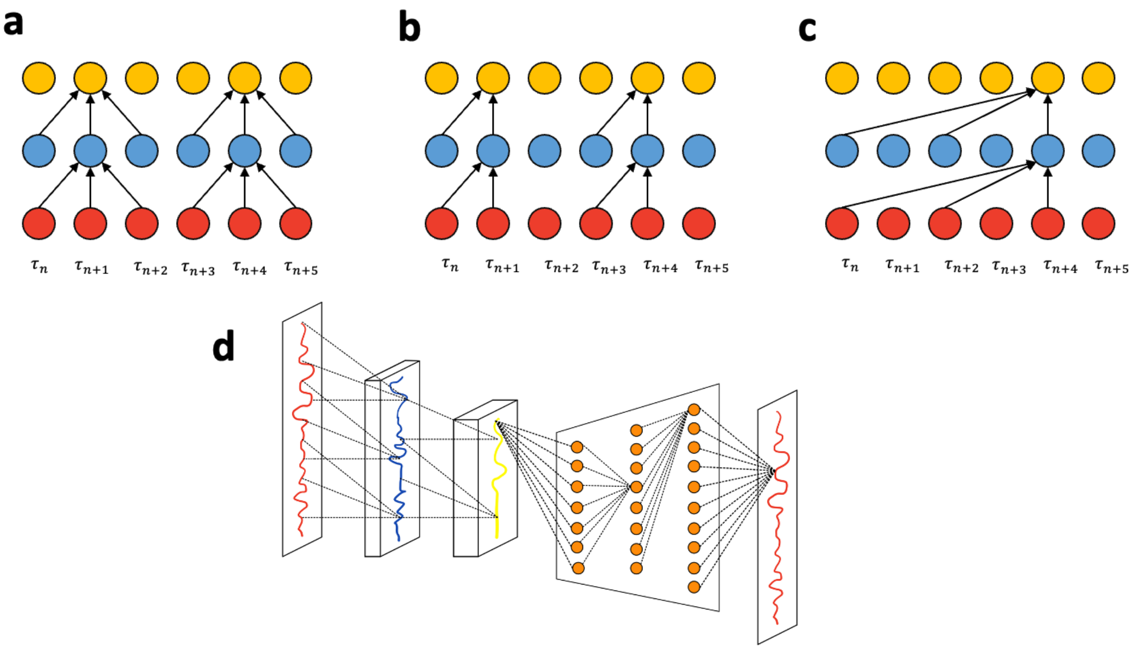

Supplementary Fig. 1. Causal convolutional autoencoder architecture

(A) Classical convolutional neural network. In a standard convolutional neural network, 1D convolution layers compute local weighted averages of subsequences within the time series, considering both information from future and past time points to generate representations corresponding to the central time point of the subsequence in the next layer.

(B) Causal convolutional neural network. A causal convolutional neural network imposes an additional constraint that representations of time points in later layers are constructed using only convolutions of past time points.

(C) Diluted causal convolutional neural network. Adding “dilution” to a convolutional layer requires representations to be from every *D* time points in the past instead of the most recent points. *D* is the dilution factor. The dilution factor is gradually increased with added layers to compute representations that integrate dynamical information at various time scales.

(D) Hybrid causal convolutional encoder-multilayer perceptron decoder. Our autoencoder uses a diluted causal convolutional encoder with a multilayer perceptron (MLP) for the decoder. The causal encoder allows effective representation of the time-series nature of our input; the MLP does not impose time-structure constraint on the embeddings during decoding.

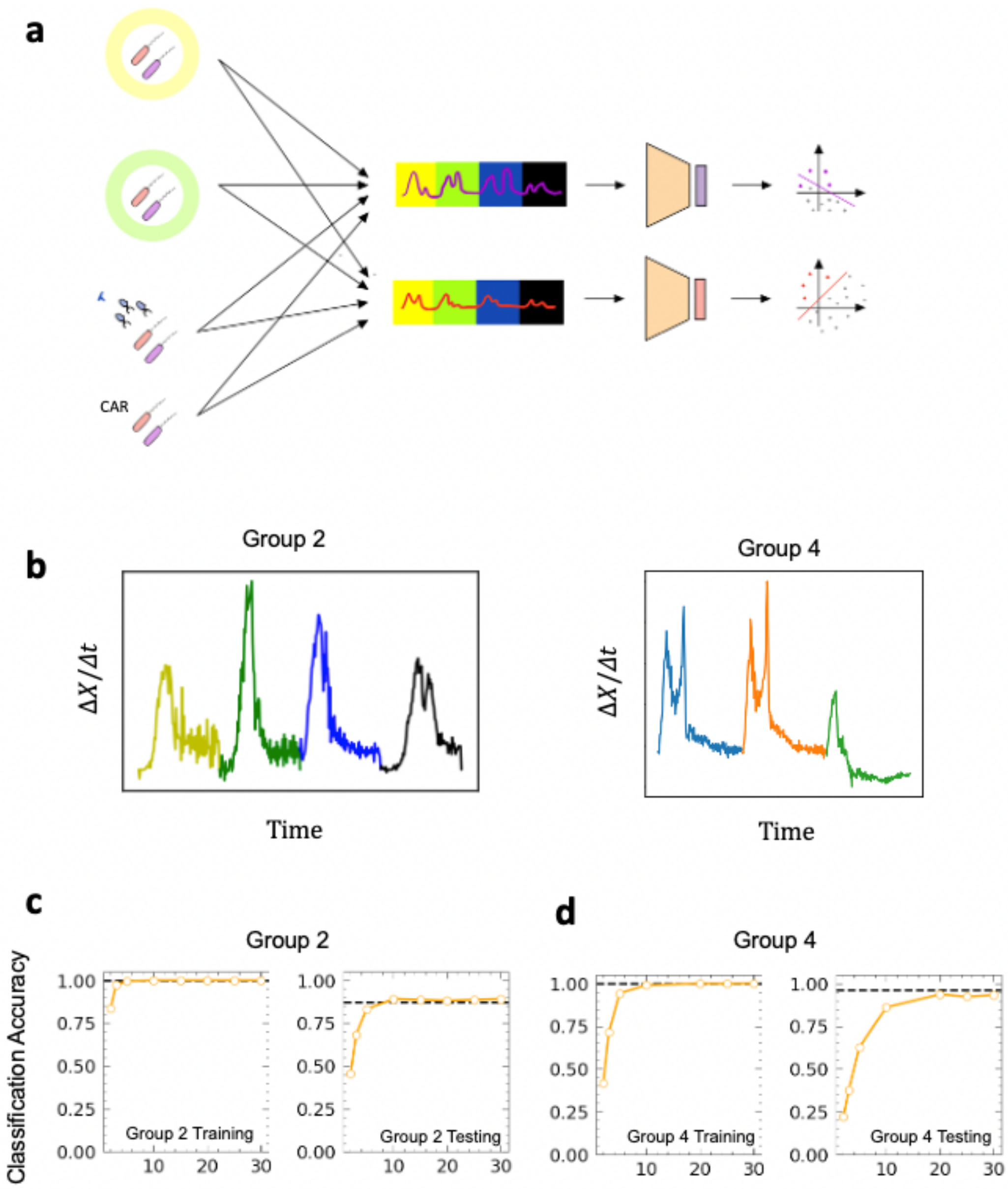

Supplementary Fig. 2. Classifying bacterial strains using concatenated curves

(A) Procedure for classifying growth curves generated from multiple experimental conditions. The time derivatives of growth curves corresponding to the same strain at different growth conditions (see Methods) are concatenated into a single time series. These concatenated curves are used to train an autoencoder and subsequently SVMs for classification.

(B) Examples of concatenated time courses for two separate groups. In group one, 244 clinical isolates were grown in four conditions (100x Luria Broth Dilution, 10,000x Luria Broth Dilution, Lambda Phage, and Carbenicillin).[1] Each concatenated curve consists of 4 (yellow, green, blue, black) growth curves. In group two, 311 additional isolates were grown in three conditions, each with different amounts of antibiotic concentration (pure LB, 50 μg/mL

amoxicillin, 50 μg/mL amoxicillin + 25 μg/mL clavulanic acid). Each concatenated curve consists of 3 (blue, orange, green) growth curves.

(C) Classification accuracy from concatenated curves in group 2. As the embedding dimension of the autoencoder increases, the classification accuracy rapidly increases before reaching asymptote for both training and testing sets. On the training set, the classification accuracy using the embedding matches (at $E < 10$) that using the full time series. On the testing set, the classification accuracy using the embedding marginally outperforms (group 2, at $E = 10$) that using the full time series.

(D) Classification accuracy from concatenated curves in group 4. As the embedding dimension of the autoencoder increases, the classification accuracy rapidly increases before reaching asymptote for both training and testing sets. On the training set, the classification accuracy using the embedding matches (at $E = 10$) that using the full time series. On the testing set, the classification accuracy using the embedding approaches (at $E = 20$) that using the full time series with $D = 432$, representing an approximately 20-fold compression.

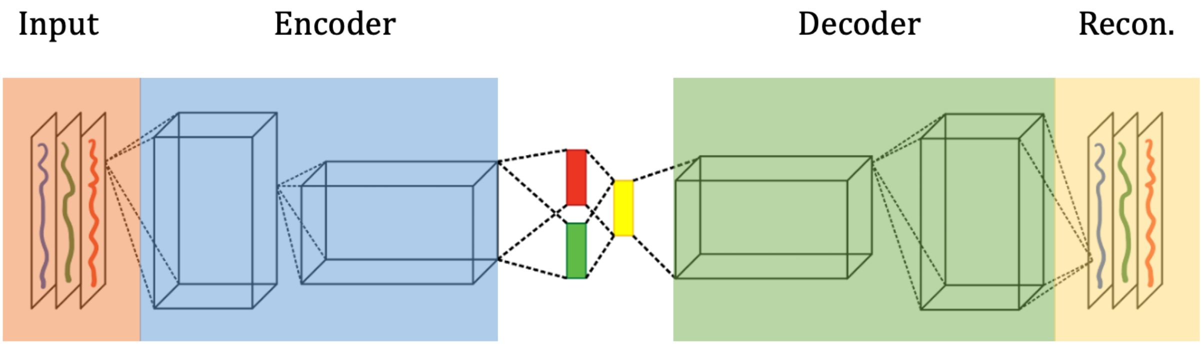

Supplementary Fig. 3. Schematic of convolutional variational autoencoder. The encoder component of the network starts with of a series of convolutional operations that generate multiple filters per dataset, represented with the boxes. As the information propagates down the network, the number of filters increases but the size of each filter decreases. At the end of the convolutional segment of the encoder, the features are flattened into a single vector and mapped to two separate equal-dimensional latent representations. One is the mean vector $\boldsymbol{\mu}$ (red) and the other is the standard deviation vector $\boldsymbol{\sigma}$ (green). The two vectors define a multidimensional Gaussian, which is used to associate a small region of latent space with each training example.

A point can be sampled using these latent features by computing $\mathbf{l} = \boldsymbol{\mu} + \boldsymbol{\sigma}\,\boldsymbol{\epsilon}$ where $\boldsymbol{\epsilon}_{\mathrm{i}} \sim N(0,1)$ is a Gaussian random vector. The sample vector is passed through another dense linear layer to generate the final latent representation $\mathbf{z}$ (yellow). $\mathbf{z}$ is passed through a decoder network, which is symmetric to the encoder network, to generate a reconstruction. During training, the network aims to minimize both the reconstruction loss between the initial and final growth curves as given by the mean square error and the KL divergence between the learned latent distribution and standard Gaussian. The former loss ensures that the network learns latent vectors with high information embedding of the high dimensional growth curve distribution while the second loss ensures that the overall distribution of latent embeddings remains continuous to enable interpolation.

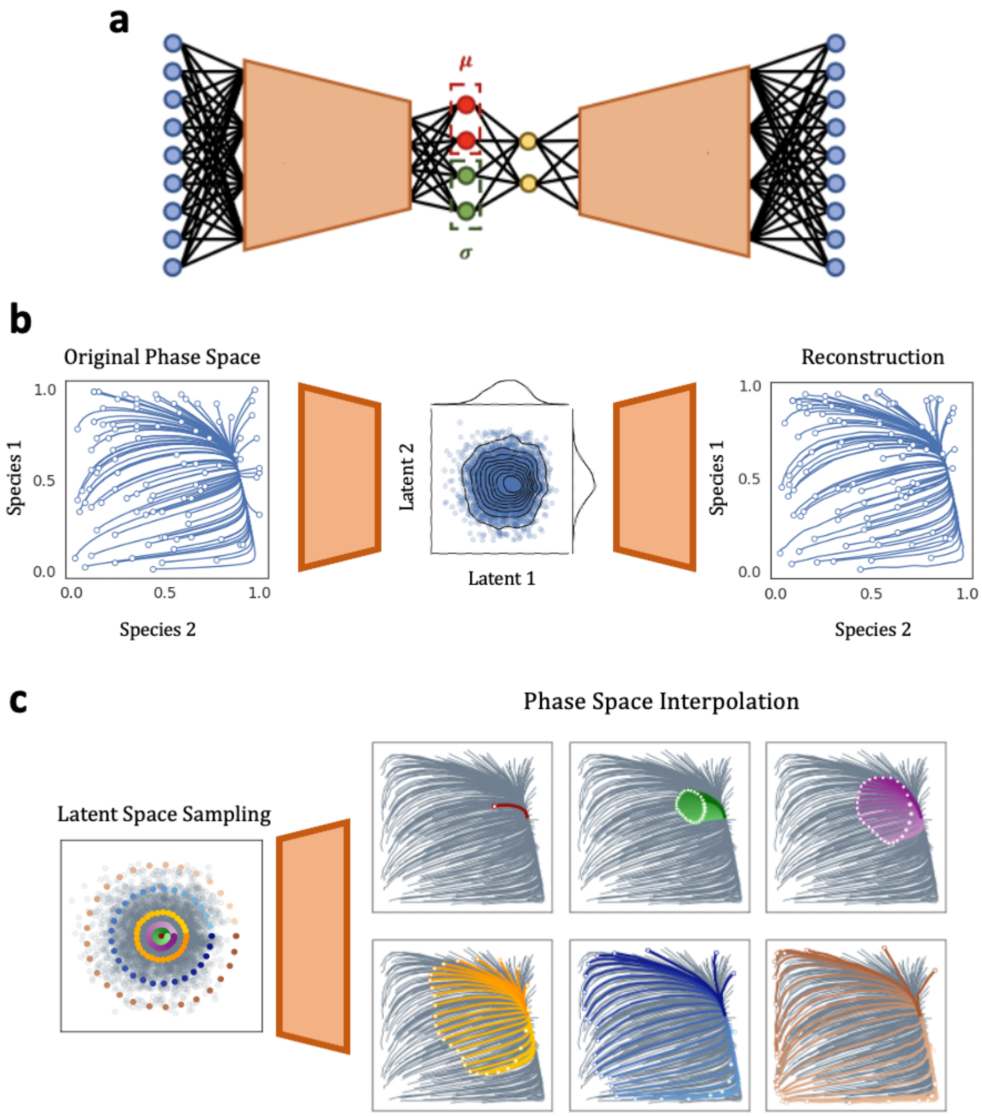

Supplementary Fig. 4. A VAE generates low dimensional, interpolatable latent spaces.
(A) VAE architecture. A VAE compresses input data to low dimensional representations but regularizes the distribution of latent embeddings into a Gaussian. This is achieved during training by learning embeddings which simultaneously minimize both the reconstruction loss, to ensure high quality reconstructions, and a Kullbeck-Leibler divergence term that quantifies the discrepancy of the distribution of data points from a standard Gaussian.

(B) Gaussian embedding of simulated two-member microbial community dynamics. 4,800 pairs of growth curves were used to train a variational autoencoder. Each training example corresponded to two 30-point growth curves generated from Eq 2, starting from a unique initial condition. The parameters of the LV model were chosen at random and fixed for all simulations. The resulting phase-space of gLV simulation is shown on the left, its two-dimensional VAE embedding in the center, and the reconstructed phase space on the right. The circles indicate the initial condition for a trajectory. Kernel density estimation of the latent distribution revealed creation of the latent embeddings with a continuous, Gaussian-like unimodal distribution.

(C) Latent-space interpolations reveal two-dimensional polar parametrization of phase space. We sampled and decoded points in the latent space in six sets of rings, each made of points with the same radius from the center of the Gaussian distribution. The left panel shows the points in the latent space corresponding to training data in gray and the novel sample points in color. The corresponding interpolated trajectories in the reconstructed phase space are indicated on the right with using the same color. As distance from the center of the latent distribution increased, so does arclength distance from the fixed point in the phase space. At a fixed radius, varying the angular position of a sample point about the mean of the Gaussian similarly varies the angular position of the initial position about the fixed point of the corresponding interpolated trajectory. This suggests the VAE achieves low dimensional embedding by learning a polar coordinate system about the fixed point of the dynamics.

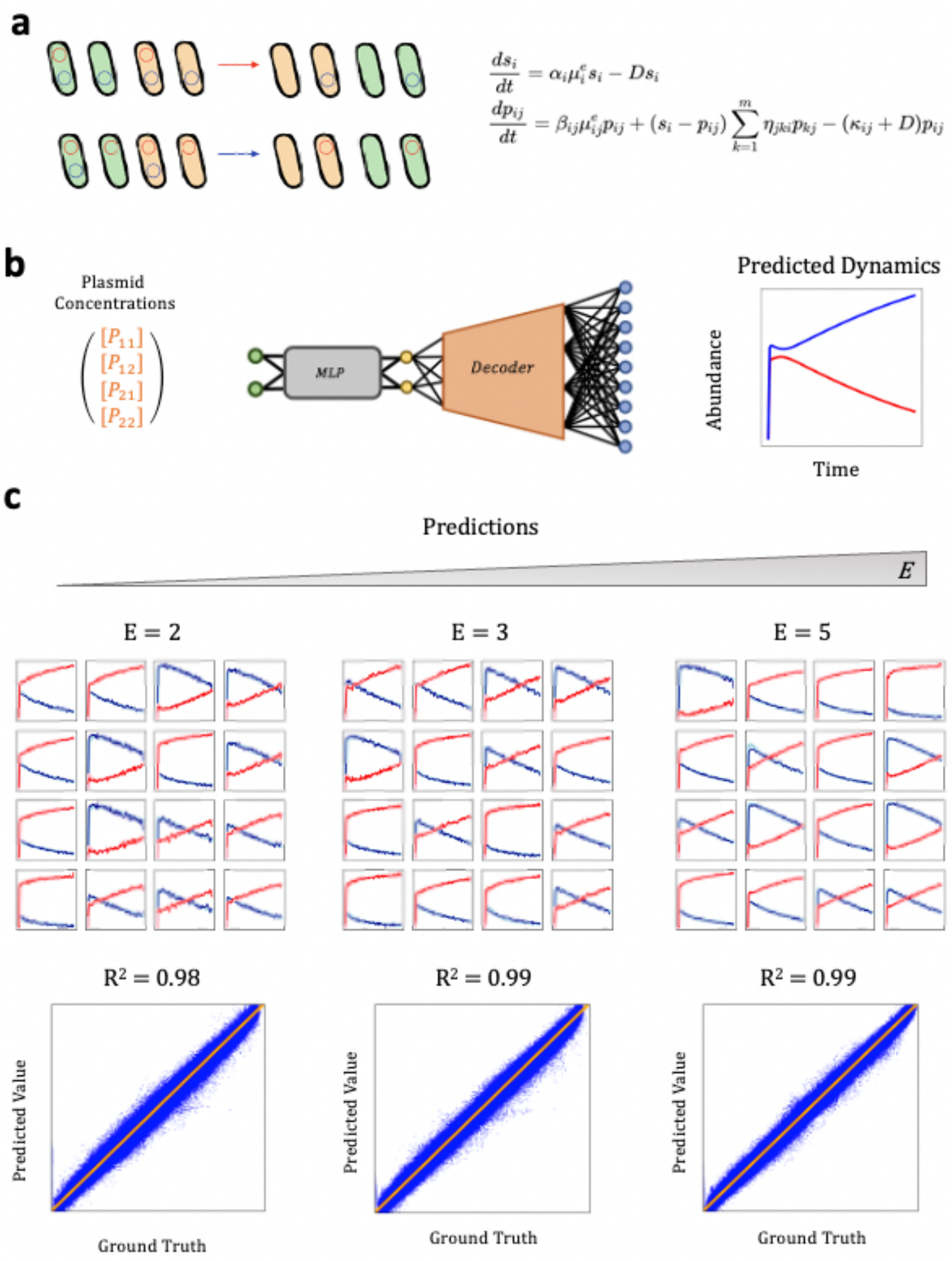

Supplementary Fig. 5. VAE enables dynamics prediction for communities with HGT.
(A) Horizontal gene transfer model. We simulate the dynamics of a two-member microbial community trading two plasmids using a plasmid centric framework. Briefly, we separately simulate the population dynamics of the individual species i and within each species, the population carrying a given plasmid j. The resulting system is thus captured by 2 ODEs describing the species dynamics and 4 ODEs corresponding to the dynamics of plasmids being carried within each population (Methods). Using this formulation, we generate a dataset of 5000 community growth curves by varying the initial concentration of each plasmid while holding all other system parameters and initial conditions fixed.

(B) Two step initial condition-to-dynamics mapping. As with the other prediction problems, our procedure consists of first training a variational autoencoder to learn a low dimensional embedding of our system dynamics. A multilayer perceptron is then used to predict the species population dynamics from initial distribution of plasmids within the species. Note, though our ODE model resolves the dynamics of the both the species and plasmids, we train our variational autoencoder using only species dynamics, so the model has no explicit information on the dynamics of horizontal gene transfer except through their implicit impacts on population dynamics.

(C) VAE-MLP enables high accuracy prediction of community dynamics. Despite not having any information on the plasmid dynamics, we see the VAE-MLP can predict the community dynamics of both species from initial plasmid concentration to high accuracy even at very low dimension of $E = 2$. As the embedding dimension improves, the quality of prediction increases, though slightly.

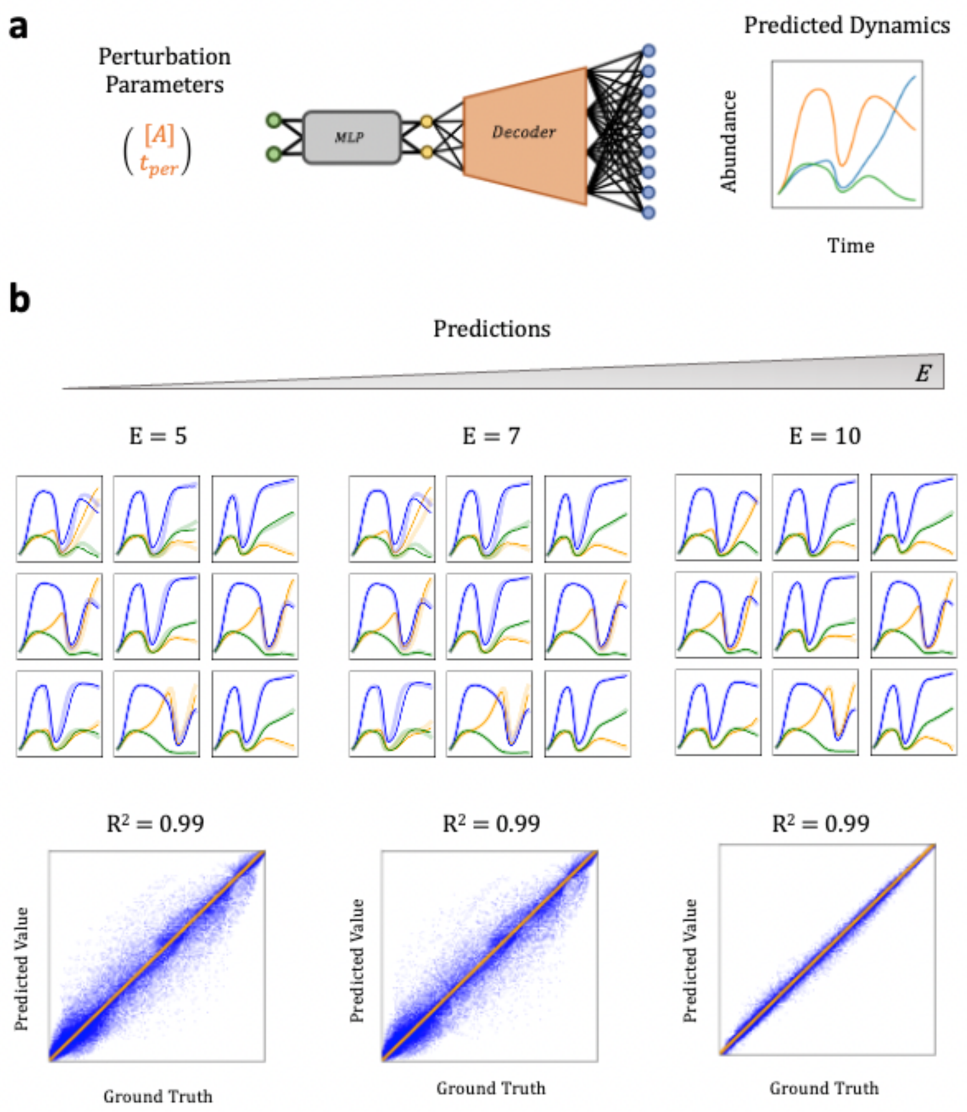

Supplementary Fig. 6. VAE enables prediction of growth dynamics resulting from temporal perturbations.

(A) Mapping temporal perturbations to dynamics. We generated a dataset of 5000 simulated 3-member community dynamics using a gLV model. Each sample consists of growth curves (each of 50 data points) of the 3 members. The model parameters and initial compositions were fixed for all simulations. The community dynamics were perturbed by an antibiotic added at variable doses and time points. To predict community dynamics, as with the initial-condition-to-trajectory model, we first train a VAE on the community dynamics to generate a low dimensional latent space to represent these dynamics. We then train an MLP that maps the parameters of the antibiotic perturbation (antibiotic dosage and time of perturbation) to a corresponding point in the latent space, which is in turn decoded to get a prediction of the community dynamics.

(B) VAE-MLP enables high accuracy prediction of growth dynamics from temporal perturbations. Even with a low latent dimension of $E = 5$, the MLP-VAE mapping predicted community dynamics with a high accuracy. As the embedding dimension is increased, the accuracy improves as evidenced by the shrinking scatter about the line $y = x$. Here we show the predictions in the test dataset.

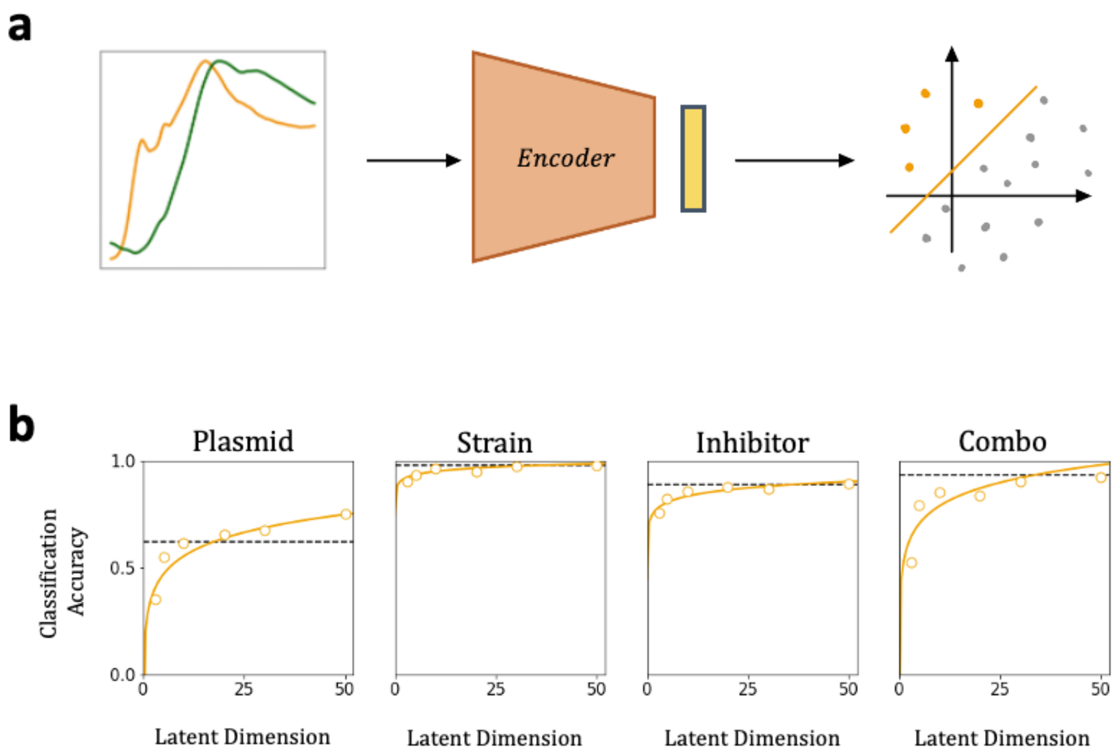

Supplementary Fig. 7. Classifying phenotypes from 2-member community dynamics
(A) Procedure for classifying two-member community dynamics. As with previous datasets, we concatenate together our two readouts of community dynamics (GFP and OD). These concatenated curves (290 total time points) are then used to train a variational autoencoder. The subsequent embeddings of the curve and then fed into a SVM for final phenotype classification.

(B) A common latent space enables accurate prediction of multiple environmental and phenotypical properties. Our dataset consists of 7200 individual population time courses corresponding to 1200 community dynamics experiment conditions. In each experiment, one strain carried a plasmid conferring both fluorescence and resistance to a beta-lactam antibiotic while the other lacked any plasmid and was sensitive to antibiotic. Across the experiments we fixed the initial population of both strains but varied various other conditions including the identity of the bacterial strain (DA28102 vs Top10F), the type of plasmid carried by the resistant strain (Bla vs BlaM vs HSGBla vs HSGBlaM), and the identity of beta-lactamase enzyme inhibitor (Clavulanic acid vs Sulbactam vs Tazobactam) (Methods). Using our VAE-SVM method, we aim to classify from latent embeddings the identity of strain, plasmid, inhibitor, and then each unique combination of features jointly as one label. We see as the embedding dimension increases the testing classification accuracy increases (orange), but even for relatively small values of $E = 25$, the testing classification accuracy has achieved parity or superiority relative to the classification accuracy on full growth curves (black dashed line) for all four classification tasks.

#	Description	# Strains	Samples per Strain	Features	Ref.
1	Clinical isolates sampled from North Carolina Hospitals, grown in 10,000x LB Dilution.	203	4	98	[1]
2	Same clinical isolate strains as in Group 1 but grown in four different culture conditions and then concatenated into one curve.	203	4	392	[1]
3	Environmental isolates sampled from various locations around Duke University Campus.	143	12	98	[1]
4	Clinical isolates sampled from North Carolina Hospitals, grown in LB under three different culture conditions and concatenated into one curve.	311	12	432	Methods

Supplementary Table 1. Summary of growth curve groups used in classification and resistance prediction. Each group consists of a set of growth curves corresponding to one of several distinct strains (# strains). For each strain, we have a finite set of samples corresponds to individual population growth curves of bacteria of that strain or population growth curves of bacteria of that strain grown in different culture conditions and then concatenated together (samples per strain). Each of these growth curves (or concatenated growth curves) vary in their number of total measurement time points (features). For Group 1 for example, we have 203 unique strains, 4 single growth curves per strain, and each growth curve (after finite differencing) consists of 98 time points.

Supplementary Methods

Predicting microbial community dynamics involving horizontal gene transfer

To simulate the dynamics of a two-member microbial community exchanging two plasmids, we leveraged a plasmid-centric ODE framework originally developed by Wang et. al.[2] Briefly, we separately simulate the population dynamics of the individual species i and within each species, the population carrying a given plasmid j. The resulting system is thus captured by 2 ODEs describing the species dynamics and 4 ODEs corresponding to the dynamics of plasmids being carried within each population:

$$\begin{aligned}
\frac{ds_1}{dt} &= \alpha_1 \mu_1^e s_1 - D s_1 \\
\frac{ds_2}{dt} &= \alpha_2 \mu_2^e s_2 - D s_2 \\
\frac{dp_{11}}{dt} &= \beta_{11} \mu_{11}^e p_{11} + (s_1 - p_{11})(\eta_{111} p_{11} + \eta_{121} p_{21}) - (\kappa_{11} + D)\, p_{11} \\
\frac{dp_{12}}{dt} &= \beta_{12} \mu_{12}^e p_{12} + (s_1 - p_{12})(\eta_{211} p_{12} + \eta_{221} p_{22}) - (\kappa_{12} + D)\, p_{12} \\
\frac{dp_{21}}{dt} &= \beta_{21} \mu_{21}^e p_{21} + (s_2 - p_{21})(\eta_{122} p_{21} + \eta_{112} p_{11}) - (\kappa_{21} + D)\, p_{21} \\
\frac{dp_{22}}{dt} &= \beta_{22} \mu_{22}^e p_{22} + (s_2 - p_{22})(\eta_{222} p_{22} + \eta_{212} p_{12}) - (\kappa_{22} + D)\, p_{22}
\end{aligned} \tag{1}$$

Where s_i is the population of species i and p_{ij} is the size of the population of species i carrying plasmid j. Thea α_i terms correspond to the impact on intrinsic growth rate of a species due to the combined effect of the plasmids carried by the population modulated by the prevalence of a given plasmid and its relative growth burden boon on species i given by the parameter λ_{ij}. Explicitly they are related by the weighted sum:

$$\alpha_1 = \frac{s_1}{s_1 + p_{11}\lambda_{11} + p_{12}\lambda_{12}}$$

α_2 is given similarly. The β_{ij} parameters indicate the impact on the growth of the individual plasmid carrying subpopulation due to the plasmid abundance within that population and is calculated similarly to $\alpha_i . \mu_i^e$ gives the effective growth rate and is given by the usual logistic growth term $\mu_i^e = \mu_i(1 - \sum_j s_j)$ where μ_i is the basal growth rate. The η_{ijk} terms represent the

conjugation rate of plasmid j from species k to species i. Lastly, the dilution parameter D represents loss of species due to background outflow and dilution of the culture, which is usually present in real microbial systems. Similarly, κ_{ij} represents the intrinsic loss rate of plasmid j by species i. A more detailed discussion of this model and its derivation can be found in Wang et. al.[2]

For our simulations we fix the values of $D = 0.005, \kappa_{ij} = 0.001, \mu_i = 0.3$ for all simulations. We rolled the values of η_{ijk} from a Gaussian distribution with mean 0 and variance $\sigma = 0.01$. Similarly, we drew the values of $\lambda_{ij}$from a Gaussian with mean zero and variance $\sigma = 0.5$. We took the absolute value of all these randomly drawn values afterwards to ensure positive values. To generate our dataset, we ran 5000 simulations keeping these parameters fixed and fixing the initial population of both species to $s_i = 0.04$, but randomly drawing the initial distribution of plasmids from a uniform distribution of $p_{ij}(t = 0) \sim \mathrm{Uni}(0.04)$ so at least a plasmid was not present in the population at maximum the plasmid was present across the entire population. We numerically integrated the system for 100 time points over 3000 arbitrary time units.

Predicting community dynamics experiencing transient antibiotic perturbation

To model a microbial system undergoing perturbation at a random time point, we adapted equation 2 to consider density dependent antibiotic death:

$$\frac{dp_i}{dt} = \mu_i p_i \left(1 - p_i - \frac{\sigma}{1 + \sum p_j \gamma_{ij}^{+}} - \sum p_j \gamma_{ij}^{-}\right) - \kappa_i a p_i \tag{2}$$

Where a represents the concentration of antibiotic in the system and k_i the killing rate of the antibiotic for species i. For the dynamics of the antibiotic, we assume they obey a simple exponential decay:

$$\frac{da}{dt} = -Da$$

Where D is a fixed antibiotic decay rate. During the simulation we assume that antibiotic is gradually added to the system at double the decay rate before the addition is terminated at some point t_0, where the antibiotic reaches its maximal concentration of a_0. After this timepoint, the antibiotic simply decays at rate D. The dynamics of the antibiotic are thus given by:

$$a(t) = a_0\, e^{2Dt}\big(1 - \Theta(t - t_0)\big) + a_0\, e^{-Dt}\Theta(t - t_0)$$

where $\Theta(x)$ is the usual Heaviside step function.

During simulations, we fixed the system parameters for all values except for a_0 and t_0, which were instead drawn randomly from simulation according to $a_0 \sim \mathrm{Uni}(1,2)$ and $t_0 \sim \mathrm{Uni}(10,30)$. We fixed the value of the ecological growth parameters as with prior simulations as $\sigma = 0.05$, $\kappa_i = -1, D = 0.5,$ and $p_i(t = 0) = 0.05$. We fixed the values of μ_i and γ_{ij} randomly as:

$$\mu = \begin{pmatrix} 0.54 \\ 0.67 \\ 0.64 \end{pmatrix} \text{ and } \gamma = \begin{pmatrix} 0.02 & 0.14 & -2.4 \\ -0.55 & 1.53 & 1.18 \\ -2.16 & 0.77 & -0.55 \end{pmatrix}$$

We integrate our system for fifty timepoints over 40 arbitrary time units for each simulation.

Supplementary References

1 Zhang, C. *et al.* Temporal encoding of bacterial identity and traits in growth dynamics. *Proc Natl Acad Sci U S A* **117**, 20202-20210 (2020). https://doi.org:10.1073/pnas.2008807117

2 Wang, T. & You, L. The persistence potential of transferable plasmids. *Nat Commun* **11**, 5589 (2020). https://doi.org:10.1038/s41467-020-19368-7
