# Supplementary material for: Autoencoder neural networks enable low dimensional structure analyses of microbial growth dynamics
Source: Nat Commun. 2023 Dec 1;14:7937. doi: 10.1038/s41467-023-43455-0 (PMC10696002; doi:10.1038/s41467-023-43455-0)
Supplement: Supplementary file 3 — Reporting summary [file 41467_2023_43455_MOESM3_ESM.pdf]

nature portfolio

Corresponding author(s): Lingchong You

Last updated by author(s): November 1, 2023

Reporting Summary

Nature Portfolio wishes to improve the reproducibility of the work that we publish. This form provides structure for consistency and transparency in reporting. For further information on Nature Portfolio policies, see our Editorial Policies and the Editorial Policy Checklist.

Statistics

For all statistical analyses, confirm that the following items are present in the figure legend, table legend, main text, or Methods section.

n/a	Confirmed	
x	☐	The exact sample size (*n*) for each experimental group/condition, given as a discrete number and unit of measurement
x	☐	A statement on whether measurements were taken from distinct samples or whether the same sample was measured repeatedly
x	☐	The statistical test(s) used AND whether they are one- or two-sided<br>*Only common tests should be described solely by name; describe more complex techniques in the Methods section.*
x	☐	A description of all covariates tested
x	☐	A description of any assumptions or corrections, such as tests of normality and adjustment for multiple comparisons
x	☐	A full description of the statistical parameters including central tendency (e.g. means) or other basic estimates (e.g. regression coefficient) AND variation (e.g. standard deviation) or associated estimates of uncertainty (e.g. confidence intervals)
x	☐	For null hypothesis testing, the test statistic (e.g. *F*, *t*, *r*) with confidence intervals, effect sizes, degrees of freedom and *P* value noted<br>*Give P values as exact values whenever suitable.*
x	☐	For Bayesian analysis, information on the choice of priors and Markov chain Monte Carlo settings
x	☐	For hierarchical and complex designs, identification of the appropriate level for tests and full reporting of outcomes
x	☐	Estimates of effect sizes (e.g. Cohen's *d*, Pearson's *r*), indicating how they were calculated

Our web collection on statistics for biologists contains articles on many of the points above.

Software and code

Policy information about availability of computer code

Data collection	No software was used for data collection in this study
Data analysis	All data analysis was conducted with custom code written in Python 3.11 using NumPy 1.25, SciPy 1.11, and Matplotlib 3.8.1 python ilbraries. Neural network training was performed using PyTorch 2.1.0. SVM fitting was performing using the implementation in scikit-learn 1.3.0 library. All code is available at https://github.com/yasab27/LSMGD.

For manuscripts utilizing custom algorithms or software that are central to the research but not yet described in published literature, software must be made available to editors and reviewers. We strongly encourage code deposition in a community repository (e.g. GitHub). See the Nature Portfolio guidelines for submitting code & software for further information.

Data

Policy information about availability of data

All manuscripts must include a data availability statement. This statement should provide the following information, where applicable:

- Accession codes, unique identifiers, or web links for publicly available datasets
- A description of any restrictions on data availability
- For clinical datasets or third party data, please ensure that the statement adheres to our policy

All data and code used in the in the main text and the supplementary information is available at: https://github.com/yasab27/LSMGD

Research involving human participants, their data, or biological material

Policy information about studies with human participants or human data. See also policy information about sex, gender (identity/presentation), and sexual orientation and race, ethnicity and racism.

Reporting on sex and gender	N/A
Reporting on race, ethnicity, or other socially relevant groupings	N/A
Population characteristics	N/A
Recruitment	N/A
Ethics oversight	N/A

Note that full information on the approval of the study protocol must also be provided in the manuscript.

Field-specific reporting

Please select the one below that is the best fit for your research. If you are not sure, read the appropriate sections before making your selection.

- [x] Life sciences
- [ ] Behavioural & social sciences
- [ ] Ecological, evolutionary & environmental sciences

For a reference copy of the document with all sections, see nature.com/documents/nr-reporting-summary-flat.pdf

Life sciences study design

All studies must disclose on these points even when the disclosure is negative.

Sample size

The temporal dynamics of every configuration of 2-member microbial communities analyzed in this study were measured in triplicate (n = 3). For growth curves used in Group 1 and 2 , we used n=4 samples per strain. For growth curves in datasets 3 and 4 , we used n=12 samples per strain.

The sample sizes were chosen based on the scale of the project and consistency with other similar published studies. For growth curves from Groups 1 and 2, the data was taken from [1]. Here for each strain, each experiment was conducted in quadruplicate with three samples used to in the training set and one sample used in their test set. To compare our AE-based methods results to their raw-data-based analysis, we chose the same datasets and sample split ratio between train and test set sizes to enable direct comparison. Similarly, for Group 3 dataset, which was also taken from [1] we used a 3:1 split ratio of their twelve samples per class to enable direct comparison to their results. For the data generated in group 4, we collected 12 samples per clinical strain following to match with the highest amount of data per class used in [1]. For the dataset of the 2-member microbial communities, we measured each experimental configuration in triplicate based on the sample sizes used in [1] to account for experimental variability in measuring growth dynamics for a given condition while also being able to screen a wide range of experimental conditions (i.e. antibiotic concentrations) in high throughput.

[1] Zhang, C. et al. Temporal encoding of bacterial identity and traits in growth dynamics. Proc Natl Acad Sci U S A 117, 20202-20210 (2020). https://doi.org:10.1073/pnas.2008807117

Data exclusions

No data was excluded from the course of the study.

Replication

Each experiment and simulation was conducted at least three times, suggesting the robustness of our analysis.

Randomization

For splitting data into training and testing sets for various machine learning tasks, we used either 2:1 or 3:1 ratio with samples allocated between training and testing classes at random. For hyperparameter tuning in the SVMs, we leveraged randomized 3-fold cross validation, which splits the training set further into three random partitions. Two partitions are used to train the model while the the third is used to evaluate the performance of a given HP set. This procedure is repeated three times, swapping the hold-out evaluation partition on each try, and at the end a single accuracy metric is computed by averaging performance across all three random partitions.

Blinding

No group allocation was involved in this study. Knowledge of a samples identity did not affect the experimental conclusion.

Reporting for specific materials, systems and methods

We require information from authors about some types of materials, experimental systems and methods used in many studies. Here, indicate whether each material, system or method listed is relevant to your study. If you are not sure if a list item applies to your research, read the appropriate section before selecting a response.

Materials & experimental systems

n/a	Involved in the study
☒	☐ Antibodies
☒	☐ Eukaryotic cell lines
☒	☐ Palaeontology and archaeology
☒	☐ Animals and other organisms
☒	☐ Clinical data
☒	☐ Dual use research of concern
☒	☐ Plants

Methods

n/a	Involved in the study
☒	☐ ChIP-seq
☒	☐ Flow cytometry
☒	☐ MRI-based neuroimaging
